# Neutrophil-lymphocyte ratio in the early diagnosis of sepsis in an intensive care unit: a case-control study

**DOI:** 10.5935/0103-507X.20190010

**Published:** 2019

**Authors:** Eduarda Cristina Martins, Lilian da Fe Silveira, Karin Viegas, Andrea Diez Beck, Geferson Fioravantti Júnior, Rafael Viegas Cremonese, Priscila Schmidt Lora

**Affiliations:** 1 Hospital Mãe de Deus - Porto Alegre (RS), Brasil.; 2 Universidade do Vale do Rio dos Sinos - Porto Alegre (RS), Brasil.

**Keywords:** Sepsis/diagnosis, Blood cell count, Clinical laboratory techniques, Lymphocyte count/methods, Neutrophils, Intensive care units

## Abstract

**Objective:**

To evaluate the neutrophil-lymphocyte ratio as a predictor of sepsis and mortality in patients admitted to an intensive care unit.

**Methods:**

Case-control study of adult patients admitted to an intensive care unit. Patients who had sepsis as the reason for admission and who had a previous complete blood count examination were included as case patients. The following statistical analyses were performed: ROC curves, binary logistic regression, and Mann-Whitney and Pearson's chi-square tests. p < 0.05 was considered significant.

**Results:**

The ROC curve values were 0.62 for neutrophil-lymphocyte ratio, 0.98 for band neutrophils and 0.51 for total leukocytes. The presence of a neutrophil-lymphocyte ratio greater than 5.0, leukocyte count above 12,000mm^3^/mL and band neutrophil percentage above 10% were risk factors for sepsis; however, only the SAPS 3 and SOFA score were related to patient mortality.

**Conclusion:**

The neutrophil-lymphocyte ratio and band neutrophils in combination with other parameters may be markers for the early detection of sepsis in intensive care units.

## INTRODUCTION

Sepsis is the presence of life-threatening organic dysfunction caused by a dysregulated response of the body to infection. Worsening of this condition leads to septic shock, which is characterized by severe circulatory and/or metabolic abnormalities, enough to cause death.^(^^1^^)^

Sepsis is one of the main causes of hospitalization and mortality in adult intensive care units (ICUs). Worldwide, it is estimated that 19.4 million patients develop sepsis per year, of which 14.1 million survive hospitalization. In Brazil, the mortality associated with septic shock is over 60%, and the world average is approximately 37%.^(^^2,3^^)^

Early diagnosis of sepsis is essential for reducing the high morbidity and mortality rate in these patients.^(^^4,5^^)^ However, sepsis is often diagnosed late because the signs and symptoms used, such as change in leukocyte count, fever, tachycardia, and tachypnea, are nonspecific and are not always present.^(^^6^^)^

There are several biomarkers that have already been studied for the early diagnosis of sepsis. These markers can be divided into risk prediction, diagnosis, monitoring and outcome.^(^^7,8^^)^ In this group of molecules, we can highlight the success of some, such as procalcitonin and CD14,^(^^7,9^^)^ but they are costly and not feasible options for low- and middle-income countries, such as Brazil.

The neutrophil-lymphocyte ratio (NLR) is an inflammatory biomarker that can be used as an indicator of systemic inflammation; the NLR is defined by the absolute number of neutrophils divided by the absolute number of lymphocytes. It is a simple measure that does not add costs to complete blood count laboratory examinations, which are performed routinely in hospitals. The NLR has been tested as a guide for the prognosis of various diseases, such as cancer, community pneumonia and sepsis.^(^^10-13^^)^

The objective of this study was to evaluate the ability of the NLR to predict early sepsis diagnosis and mortality and to describe the leukocyte profile of patients admitted to an ICU.

## METHODS

This research consisted of a case-control study of patients admitted to the adult ICU of a large private hospital in the capital of the state of Rio Grande do Sul from January 2017 to December 2017. Manuscript preparation followed the STROBE recommendations.^(^^14^^)^

The evaluated hospital is certified by the *Organização Nacional de Acreditação* (ONA) and rated excellent by the Joint Commission International (JCI). At the time of the research, the adult ICU had a total of 44 beds, which were divided into five different physical areas. Despite the pre-established divisions, patients could occupy vacancies in different physical areas; thus, patients with a diagnosis of sepsis could be admitted to any of the units.

Inclusion criteria were 18 years old or older, complete blood count up to 24 hours before admission to the ICU, and registered in the computerized system of the hospital. Patients who came from the surgical department or were transferred from another hospital were excluded.

The ICU admission ratio was used to define the case and control groups. Patients with a diagnosis of sepsis - which was defined according to the institutional protocol based on the time of collection in the criteria described by Bone et al. - were included in the case group.^(^^15^^)^ Patients with other diagnoses that did not involve infection were included in the control group. Convenience sampling was used to obtain cases that presented the necessary criteria for inclusion in the study.

The ratio of individuals in the control group to individuals in the case group was 1:1. Only patients from the control group were randomized; this group included patients without a diagnosis of sepsis or with a non-sepsis infection (n = 1,488). The patients with sepsis were recruited from the emergency room, ward, and semi-intensive unit. This strategy was used to avoid bias in the study resulting from different characteristics due to the department of origin. This criterion was used to reduce the heterogeneity between groups. Randomization was based on a computerized system, Randomizer Research,^(^^16^^)^ taking into account the department of origin of the patients. This criterion aimed to homogenize the different characteristics of the groups.

Demographic and clinical data, severity scores, and outcomes were obtained from the Epimed data management system (Epimed Solutions, Brazil),^(^^17^^)^ and the laboratory results were obtained from the MV System (MV Sistemas MV Informática Nordeste Ltda.)/*Prontuário Eletrônico do Paciente* (Electronic Patient Record; PEP), version SMA-PEP.02.070.1. Data collection by the Epimed system shows a collection integrity of 99.1% according to the system data; thus, even with a retrospective research design, the data have high reliability. This study was approved by the local ethics committee (CAAE 58235016.5.3001.5328).

All laboratory tests were performed according to the protocols of the local laboratory. Complete blood counts were performed using a ROCHE XN, lactate was determined using a ROCHE C-501, and arterial blood gas tests were performed using a ROCHE ABL-800.

To assess sepsis severity in the patients, we used the Simplified Acute Physiology Score III (SAPS 3), the Sequential Organ Failure Assessment (SOFA) and the Charlson Comorbidity Index, calculated by the Epimed system.^(^^18^^)^ Outcomes were defined as unit discharge or death.

### Statistical analysis

For statistical analyses, Statistical Package for Social Science version 20.0 (SPSS, Chicago, Illinois, USA) and MedCalc Statistical Software version 16.8 (MedCalc Software, Ostend, Belgium) were used. Categorical variables are presented as absolute and relative frequencies and were analyzed using Pearson's chi-square test; quantitative variables are presented as means and standard deviations or medians and interquartile ranges and were evaluated by Student's *t* test or the Mann-Whitney test; for quantitative variable correlations, the Pearson correlation was used. Statistical significance was set at 0.05. The Kolmogorov-Smirnov test was used to determine the normality of the data. Odds ratios (OR) were used to determine risk factors.

The diagnostic and prognostic value of the variables studied for the prediction of sepsis or death were determined using receiver operating characteristic (ROC) curves. ROC curves are capable of presenting specificity and sensitivity ranging from 0.5 to 1.0. Higher values show greater power in discriminatory outcomes (sepsis/non-sepsis or discharge/death). To calculate the sensitivity and specificity values, the cutoff point for the NLR was 5.0, as proposed by Gürol^(^^19^^)^ and confirmed by the ROC curve data; for the band neutrophil and total leukocyte calculations, systemic inflammatory response syndrome (SIRS) criteria were used.^(^^20^^)^ Logistic regression analyses were performed to examine separately the association between unfavorable outcomes and each outcome (sepsis/non-sepsis or discharge/death). The inclusion and exclusion criteria were enforced in this procedure.

## RESULTS

In the year 2017, 1,922 patients were admitted to the ICU. Of these, 353 had a diagnosis of sepsis on admission, and 226 had a complete blood count laboratory examination requested within 24 hours before admission. Thus, 226 patients were included in the case group and 226 in the control group. In the control group, 21 individuals were admitted to the unit due to respiratory conditions that were not related to sepsis; these conditions were acute respiratory failure in 52% (11/21), decompensated chronic obstructive pulmonary disease in 28% (6/21), subglottic stenosis in 0.05% (1/21), pulmonary hemorrhage in 0.05% (1/21), and acute respiratory distress syndrome in 0.05% (1/21). The demographic data for the case and control groups are presented in table 1. Readmission to the unit was a factor associated with a higher prevalence of sepsis (OR 2.0; 95% confidence interval - 95%CI 1.2 to 3.4).

**Table 1:** Demographic data for cases and controls admitted to the intensive care unit

Variables	Sepsis (cases) (n = 226)	Other diagnoses (controls) (n = 226)	p value
Age (years)	76 (14)	73 (16)	0.20
Sex			0.11
Female	116 (50.2)	122 (52.8)	
Male	115 (49.8)	109 (47.2)	
Origin of admission			0.12
Emergency room	103 (44.6)	112 (48.5)	
Ward	88 (38.1)	91 (39.4)	
Semi-intensive unit	40 (17.3)	28 (12.1)	
Reasons for admission			
Sepsis infection	226 (100)		
Nosocomial pneumonia	82 (35.5)		
Community pneumonia	51 (22.1)		
Urinary tract infection	32 (13.9)		
Infection without defined source	17 (7.4)		
Abdominal infection	15 (6.5)		
Bloodstream infection	14 (6.1)		
Other infectious sources	20 (8.4)		
Neurological		54 (23.4)	
Gastrointestinal		21 (9.1)	
Respiratory		21 (9.1)	
Other		66 (28.3)	
SAPS 3	70.8 (11.7)	58.0 (13.7)	0.009*
SOFA	5.4 (3 - 7)	3 (1 - 5)	0.000*
Charlson comorbidity index	2.4 (1 - 4)	2 (1 - 4)	0.78
Readmission	51 (22.1)	28 (12.1)	0.003*
ICU stay (days)	7 (3 - 14)	4 (2 - 7)	0.000*
Hospital stay (days)	4 (1 - 25)	3 (0 - 12)	0.002*
ICU outcome			0.001*
Discharge	166 (71.9)	192 (83.1)	
Death	65 (28.1)	39 (16.9)	

SAPS 3 - Simplified Acute Physiology Score 3; SOFA - Sequential Organ Failure Assessment Score; ICU - intensive care unit. The results expressed by mean (standard deviation), number (%) or median [25% - 75% percentile].

* Statistically significant p-value (p < 0.05).

Regarding the laboratory tests evaluated (Table 2), hematocrit and hemoglobin were lower in patients diagnosed with sepsis (p < 0.05). The total number of leukocytes (p < 0.05) was higher in the patients with sepsis than that in the control group, but the platelet concentration did not differ between groups (p = 0.29). For the leukocyte subtypes, there was a higher concentration of granulocytes (neutrophils, eosinophils and basophils) in patients with sepsis than in the controls (p < 0.001). The concentration of band neutrophils was significantly higher in patients with sepsis, as was the NLR (p < 0.001). The numbers of lymphocytes and monocytes were lower in patients with sepsis, but only the number of lymphocytes was statistically significant (p = 0.002). Other assessed parameters, lactate and blood gas parameters (pH, partial pressure of carbon dioxide - PaCO_2_, partial pressure of oxygen - PaO_2_, and bicarbonate - HCO_3_) did not differ between groups.

**Table 2:** Results of laboratory tests for cases and controls admitted to the intensive care unit

Variables	Sepsis (cases) (n = 226)	Other diagnoses (controls) (n = 226)	p value
Hematocrit (%)	31.4 (6.6)	33.4 (7.3)	0.002
Hemoglobin (g/dL)	10.1 (2.3)	11.0 (2.5)	0.001
Platelets (n/µL)	252.731 (166.000 - 320.500)	217.000 (158.000 - 295.000)	0.295
Leucocytes (n/µL)	12.400 (8.890 - 17.740)	10.190 (7.760 - 14.020)	0.049*
Total neutrophils (n/µL)	10.393 (6.451 - 15.018)	7.950 (5.313 - 11.208)	0.018*
Band neutrophils (n/µL)	9.275 (5.779 - 13.721)	929 (572 - 1.766)	0.000*
Segmented neutrophils (n/µL)	1.219 (719 - 2.349)	8.697 (5.998 - 12.488)	0.000*
Eosinophils (n/µL)	0 (0 - 64)	51 (0 - 172)	0.000*
Basophils (n/µL)	0	12 (0 - 28)	0.000*
Monocytes (n/µL)	640 (380 - 1.065)	703 (500 - 1020)	0.060
Lymphocytes (n/µL)	1.041 (571 - 1512)	1.188 (873 - 1.805)	0.002*
Neutrophil-lymphocyte ratio (n/µL)	10.7 (5.7 - 17.8)	6.5 (3.46 - 10.5)	0.000*
Lactate (g/dL)	1.7 (1.0 - 3.3)	1.8 (1.08 - 2.5)	0.694
pH	7.4 (7.3 - 7.4)	7.4 (7.3 - 7.4)	0.659
PaCO_2_	37 (31 - 46)	39 (31 - 51)	0.782
PaO_2_	96 (66 - 136)	95 (73 - 122)	0.722
HCO_3_	24.0 (19.1 - 28.7)	24.3 (20.4 - 29.5)	0.557

PaCO_2_ - partial pressure of carbon dioxide; PaO_2_ - partial oxygen pressure; HCO_3_ - bicarbonate. The results expressed as the mean (SD) or median [percentile 25-75%].

* Statistically significant p-value (p < 0.05).

Neutrophil-lymphocyte ratio, band neutrophils and total leukocytes were evaluated for sepsis prediction. The ROC curve values were 0.62 (95%CI 0.55 - 0.69) for NLR, 0.98 (95%CI 0.97 - 1.0) for band neutrophils, and 0.51 (95%CI 0.44 - 0.59) for total leukocytes. The performance of the NLR, total leucocytes and band neutrophils for sepsis prediction in ICUs is provided in table 3. Among the three parameters, the best performance was observed for band neutrophil count followed by the NLR (Table 3).

**Table 3:** Sensitivity, specificity and positive and negative predictive values for the prediction of sepsis on admission to the intensive care unit

Parameter	Sensitivity % (95%CI)	Specificity % (95%CI)	PPV % (95%CI)	NPV % (95%CI)
NLR > 5.0	81.0 (75.3 - 85.8)	35.5 (29.3 - 42.0)	55.6 (52.8 - 58.4)	65.1 (57.6 - 71.9)
Band neutrophils > 10%	98.8 (95.9 - 99.9)	63.7 (52.6 - 74.1)	85.4 (81.4 - 88.6)	96.4 (86.9 - 99.1)
Total leukocytes > 12,000	36.7 (31.6 - 42.1)	72.13 (66.7 - 77.1)	59.5 (53.9 - 64.9)	50.6 (47.9 - 53.2)
Total leukocytes < 4,000	6.9 (4 - 11)	95.2 (91.6 - 97.6)	59.3 (40.8 - 75.4)	40.6 (49.4 - 51.7)

95%CI - 95% confidence interval; PPV - positive predictive value; NPV - negative predictive value; NLR - neutrophil-lymphocyte ratio.

Neutrophil-lymphocyte ratio and band neutrophils had a weak (r = 0.2), positive and statistically significant correlation with length of hospital stay (p < 0.05); however, with time of hospitalization in the unit, a weak, positive and statistically significant correlation was found only with NLR (r = 0.3 and p < 0.05).

In a univariate logistic regression analysis, it was possible to confirm that an NLR higher than 5.0, leukocyte count above 12,000mm^3^/mL, and percentage of band neutrophils above 10% were risk factors for sepsis diagnosis (Table 4). However, none of these parameters were related to patient mortality. Only SAPS 3 and SOFA scores were related to mortality (Table 5). In a subanalysis according to patient origin, the highest ROC curve value was found for patients from the emergency room (0.70), followed by the semi-intensive unit (0.66) and the ward (0.59).

**Table 4:** Univariate binary logistic regression for the diagnosis of sepsis

Parameter	OR (95%CI)	p value
NLR > 5.0	2.4 (1.5 - 3.5)	0.000*
Band neutrophils > 10%	154 (35 - 668)	0.000*
Total leukocytes > 12,000	2.2 (1.5 - 3.24)	0.000*
Total leukocytes < 4,000	2.12 (0.96 - 4.9)	0.06*
SAPS 3	1.08 (1.062 - 1.099)	0.000*
SOFA	1.23 (1.1 - 1.3)	0.000*

OR - odds ratio; 95% CI - 95% confidence interval; NLR - neutrophil-lymphocyte ratio; SAPS 3 - Simplified Acute Physiology Score 3; SOFA - Sequential Organ Failure Assessment Score.

* Statistically significant p-value (p < 0.05).

**Table 5:** Univariate binary logistic regression for mortality at the unit for patients with sepsis

Parameter	OR (95%CI)	p value
NLR > 5.0	1.42 (0.85 - 2.38)	0.18
Band neutrophils > 10%	2.13 (1.01 - 4.50)	0.046*
Total leukocytes > 12,000	1.2 (0.77 - 1.82)	0.45
Total leukocytes < 4,000	2.5 (1.13 - 5.63)	0.023*
SAPS 3	1.077 (1.05 - 1.09)	0.000*
SOFA	1.33 (1.23 - 1.44)	0.000*

OR - odds ratio; 95% CI - 95% confidence interval; NLR - neutrophil-lymphocyte ratio; SAPS 3 - Simplified Acute Physiology Score 3; SOFA - Sequential Organ Failure Assessment Score. The laboratory parameters (neutrophil-lymphocyte ratio, band neutrophils > 10%, total leukocytes > 12,000, total leukocytes < 4,000) were measured up to 24 hours prior to admission to the intensive care unit, and severity scores were measured at admission.

* Statistically significant p-value (p < 0.05).

## DISCUSSION

Sepsis is a condition that is among the main reasons for hospitalization in the context of intensive care in Brazil.^(^^17^^)^ In SPREAD, a Brazilian multicenter study, the estimated number of beds occupied by patients with sepsis was 30.2% among patients admitted to ICUs, and the associated mortality was 55.7%.^(^^21^^)^ In the ICU evaluated, the prevalence of sepsis was 18.4%, with an associated mortality of 28%. The early detection of this condition is associated with a lower mortality rate among those affected.^(^^22^^)^

Regarding the SPREAD study,^(^^17^^)^ 58% the sample consisted of public institutions, and 66% of the institutions had high availability of resources. This reality differs from that of the evaluated hospital, which is a private hospital with international certifications, which may justify the lower prevalence of observed sepsis.

In turn, our sample was predominantly composed of elderly patients. The elderly, due to their condition, present a higher prevalence of sepsis in private hospitals, as well as severe sepsis.^(^^23,24^^)^ In a study with data from a German population, the incidence of sepsis was 12% in intensive care patients.^(^^25^^)^ In this analysis, similar to ours, the highest prevalence of intensive care patients was the elderly.

In our findings, there was no difference related to the origin of admission, with the majority of patients with sepsis coming from the emergency room or from other hospitalization units. There was also no relationship between the test performance and the origin (community or hospital) of sepsis. However, a study noted that patients from the ward had a higher risk of developing sepsis in the ICU than did patients from the emergency room.^(^^26^^)^ Readmission to the unit was associated with a greater chance of developing sepsis, which may be related to immunosuppression caused by this disease.^(^^27^^)^ As previously described,^(^^21,28^^)^ the main reasons for ICU admission were pneumonia (nosocomial or community) and urinary tract infection.

The criteria for defining sepsis have been reviewed in the literature. Parameters such as SOFA and qSOFA scores are preferred according to the definitions proposed by Sepsis-3.^(^^29^^)^ Our study was conducted in a setting that does not yet use these criteria; several other institutions do not use them as well. Although recent definitions provide good arguments for these indications, there is still controversy in the literature, in addition to the time it takes for services to assess their context and judge the need to use the proposed parameters.

SOFA and SAPS 3 scores were higher in the sepsis group than in the control group (p < 0.001), but there was no difference between groups in the Charlson index (p = 0.78). Patients who had a diagnosis of sepsis at admission to the ICU remained longer in the department, and the hospitalization time and death rate were higher in these patients. These results reinforce previous findings that sepsis is a condition associated with increased morbidity and mortality.^(^^30^^)^

Lactate is a parameter used in bundles and indicated by the Surviving Sepsis Campaign to reduce mortality in sepsis.^(^^31^^)^ There is controversy in the literature on the use of this test with an assumption of sepsis diagnosis.^(^^32^^)^ Lactate is most often associated with higher mortality and is generally described as a marker that should decrease throughout treatment; this demonstrates the success of our study, which presented laboratory results prior to hospitalization (24 hours earlier). Thus, it is justifiable that lactate and blood gas test results, which also aid in the identification of organic dysfunction, did not change because organic involvement is described as a consequence of the sepsis process.^(^^33^^)^

All parameters evaluated in the complete blood count, except for the number of platelets and monocytes, were different between the control group and the patients with sepsis. The characteristics observed in the patients were anemia, leukocytosis, neutrophilia and lymphocytopenia. Zahorec^(^^34^^)^ described, for the first time, the relationship between neutrophil and lymphocyte concentration changes in a sepsis patient. The author states in his work that patients with this condition have neutrophilia and lymphocytosis and suggests that the NLR a marker of cellular stress of the immune system in cases of infection in the context of intensive therapy.

Based on the literature and the behavior of the ROC curve generated by the data in this study, the cutoff point of 5.0^(^^19^^)^ for the NLR was associated with a high risk for sepsis, with a sensitivity greater than 80% but with low specificity. In low-income countries, such as Brazil, a low-cost test, such as a complete blood count, may contribute substantially to the early identification of sepsis, along with commonly assessed clinical criteria. In a scenario of risk for sepsis prediction, the choice for greater sensitivity is preferred to identify individuals who may develop the condition.

Recently, de Jager et al.^(^^10^^)^ studied patients with suspected community-acquired pneumonia in emergency room settings and demonstrated a significant difference between NLR values among patients with positive and negative blood cultures. A significant difference was also observed for lymphocyte concentration; however, a significant difference was not observed regarding the number of total leukocytes and total neutrophils.

In the literature, the NLR is associated not only with the early identification of sepsis but also with disease severity scores for measures such as SOFA, SAPS 3 and Acute Physiology, Age, Chronic Health Evaluation (APACHE).^(^^34,35^^)^ Hwang et al.^(^^36^^)^ showed that the NLR determined at the time of ICU admission in patients with sepsis and septic shock was associated with mortality at 28 days. In our study, it was not possible to observe the relationship with mortality when using a cutoff point of 5 for the NLR. It should be noted that the NLR data collected represented up to 24 hours prior to hospitalization, and the mortality outcome was considered in the unit, not after 28 days of hospitalization.

Zhang et al.^(^^37^^)^ evaluated 120 patients in a hospital who were referred for blood culture and had two or more SIRS criteria. They correlated NLR, total leukocytes and inflammatory parameters (C-reactive protein and prolactin) with sepsis detected by SIRS with a proven infectious outbreak or SIRS with no proven infectious outbreak. In this study, it was possible to observe that prolactin had better accuracy, followed by the NLR. Our selection did not take into consideration the request for blood culture because our objective was to evaluate the NLR as an early predictor of sepsis because it is known that the diagnosis of sepsis can occur in the absence of a positive blood culture.^(^^38^^)^ Additionally, in the health context of low- and middle-income countries, the availability of specific tests, such as that for prolactin, is not guaranteed.^(^^37^^)^

The accuracy of predicting a sepsis diagnosis in the presence of more than 10% band neutrophils is notable. This parameter is one of the criteria used in the SIRS system.^(^^20^^)^ This criterion, which includes other alterations in addition to the presence of immature neutrophils, such as tachycardia, tachypnea, leukocytosis and leucopenia, has been addressed in the literature.^(^^3,39^^)^ Regardless of whether this classification is used, this parameter is rarely used in evaluations of laboratory markers for sepsis. One reason may be related to the fact that in order to obtain these values, a manual examination using microscopy is necessary, which involves high costs with professional clinical analysts.

However, determination of the number of band neutrophils is examiner-dependent, not automated, which impacts the recommendations of the American College of Pathology regarding the number of band neutrophils and that of segmented neutrophils.^(^^40^^)^ Lawrence et al.^(^^41^^)^ concluded that flow cytometry was more accurate for determining band neutrophils in newborn infants. However, this technology is not available in most Brazilian health services as well as in other low- and middle-income countries. The accuracy tests performed in this study took into account clinical diagnoses and observable results, in addition to possible limitations resulting from manual analyses of cells.

Case-control experimental designs are described in the literature as rapid and cost-effective studies.^(^^42^^)^ Nevertheless, one strong point of this design is the ability to select an adequate number of cases in a standardized way. Considering the limitations of case-control studies, this study minimized the presence of selection biases by randomizing the selection of controls and used medical record data, which were recorded in a standardized information system by a trained team. The determination of the risk factors between the cases and controls was minimized by the use of standardized methods.

Another limitation of this study was the nondiscrimination of patients with hematological diseases or immunosuppressive drugs, which could cause alterations in the complete blood count.^(^^43^^)^

## CONCLUSION

Our study provided evidence on the use of the neutrophil-lymphocyte ratio and band neutrophils in combination with other parameters for the early detection of sepsis. The parameters presented in this study are low cost and easy to implement by health services, which reinforces their use in low- and middle-income countries, such as Brazil. Sepsis is a condition of complex physiological changes, and to ensure a reliable diagnosis, other laboratory criteria should be evaluated, such as blood culture, inflammatory markers, such as C-reactive protein or prolactin, when feasible.
